# Association between psychological distress and a sense of contribution to society in the workplace

**DOI:** 10.1186/1471-2458-12-253

**Published:** 2012-04-01

**Authors:** Kenichi Ozaki, Yutaka Motohashi, Yoshihiro Kaneko, Koji Fujita

**Affiliations:** 1 Department of Public Health, Akita University School of Medicine, 1-1-1 Hondo, Akita 010-8543, Japan

**Keywords:** mental health, job stress, psychological distress, contribution to society, bonding with workplace, meaning of work

## Abstract

**Background:**

Globally, mental health promotion related to psychological distress in the workplace has become a great concern, and a focus of much research attention. However, a sense of contribution to society and sense of bonding with the workplace have not been examined in relation to psychological distress. Thus, the purpose of this study is to examine whether these two factors are associated with psychological distress.

**Methods:**

We conducted a cross-sectional survey of 1137 full-time employees who worked in systems engineering, sales, or administration at a Japanese company. Participant's sense of contribution to society, sense of bonding with the workplace, psychological distress, and qualitative job stress (quantitative and qualitative workloads, job-control latitude, and support from supervisors, co-workers and family) were assessed with a questionnaire. We performed multiple logistic regression analyses to examine associations between psychological distress and sense of contribution to society and of bonding with the workplace.

**Results:**

A high sense of contribution to society was significantly associated with a high sense of bonding with the workplace (Spearman's ρ = 0.47, p < 0.01). A sense of contribution to society was negatively associated with psychological distress after adjusting for job stress factors (OR = 2.05, 95% CI 0.99-4.23) or sociodemographic characteristics of participants (OR = 2.92, 1.53-5.59). After adjusting for job stress factors as well as sociodemographic characteristics, the association became weaker. A sense of bonding with the workplace was negatively associated with psychological distress after adjusting for sociodemographic characteristics (OR = 2.49, 1.29-4.79). However, this association was not observed after adjusting for job stress factors.

**Conclusions:**

Psychological distress in the workplace was associated with sense of contribution to society. Therefore, workplace mental health promotion should consider the workers' sense of contribution to society.

## Background

Globally, mental health promotion related to psychological distress in the workplace has become a great concern. In Japan, there is evidence that mental illness in the workplace is increasing [1]. The ILO/WHO [2], in an investigation of five countries (Finland, Germany, Poland, the UK, and the US), suggest that the reasons for increasing numbers of mental health stressors in the workplace are complex, but are commonly related to the advancement of information technology, globalization, and rising unemployment. Japan is heavily influenced by all of these three factors.

Psychological distress is often expressed as depression, which is the most common mental illness in the workplace [3]. In recent years, depression has increased in Japan and in other countries. A national survey on current patient status in Japan conducted by the Ministry of Health, Labour and Welfare [4] shows that reported cases of physician-diagnosed major depression have increased over the last decade. There were 441 000 reported cases in 1999, 711 000 in 2002, 924 000 in 2005, and 1 041 000 in 2008, despite the population growth rate in this period being only 0.87%. Likewise, the prevalence of major depression in the United States has risen from 3.33% in 1991-92 to 7.06% in 2001-02 [5]. While the prevalence of psychiatric disorders in Asia is low, the prevalence of depression in Japan is higher than that in other Asian countries [6]. Thus, an investigation of work-related factors that influence depression in Japan is warranted.

Job stress is considered to be related to various factors, including control latitude and quantitative workload [7,8]. Karasek [9] proposes a job demand-control model of occupational stress, in which job demand latitude (the joint effects of various work stressors, including quantitative workload and role conflict), and job control latitude (the range of decision-making latitude) may affect psychological responses to stress. Individual stress levels are represented by one of four patterns that are constructed by job control level (high or low) and job demand level (high or low). The "high-strain" group comprises individuals with high job demand and low job control. This group has the highest psychological stress response and mental health risk. Johnson and Hall [10] propose a demand-control-social support model. In this model, high job demand, low control, and low social support are seen as likely to cause the most stress and health problems. Hurrell and McLaney [11] explore other indicators such as quantitative workload and role conflict as workplace stressors in their model of job stress.

Psychological wellbeing is predicted by meaningful work [12]. Jahoda [13] suggests that employment serves not only the manifest function of providing income, but also serves latent functions such as enlarging the scope of social experience into areas less emotionally charged than family life, and assignment of virtue by employment status and identity.

An annual questionnaire-based survey of business enterprises regarding the mental health of their employees' revealed the following characteristics of the current circumstances of the Japanese workplace [1]: training opportunities have decreased, opportunities to sense the whole picture and social meaning of their work have decreased, and the senses of bonding with the workplace and organization have decreased. These three characteristics were shown to have significant, positive associations with the number of employees with depression. This research was based on the views of the organization's managers and human resources managers. The views of employees themselves are yet to be established.

In modern society, workplace stress factors have become more complex [1,2]. It is known that there are relationships between psychological distress in the workplace and social support, gender [14,15], and organizational justice [16]. However, a sense of contribution to society and a sense of bonding with the workplace have not been examined in relation to psychological distress. The purpose of this study is to examine whether a sense of contribution to society and a sense of bonding with the work place are associated with psychological distress.

## Methods

The participants of this study were full-time employees in three major offices of a Japanese company located in Tokyo, Nagoya and Osaka. This company has three departments: systems engineering, sales, and administration. We conducted a questionnaire-based survey using the company intranet from November 18 to December 14, 2009. A total of 1137 employees between 20 and 59 years old were invited to participate, and responses were received from 1002, a response rate of 88.1%. Of the 1002 respondents, 937 (93.5%) had complete data. Incomplete data appeared to be due to random error.

The questionnaire included items assessing sense of contribution to society and sense of bonding with the workplace, as well as measures of psychological distress, job stress factors, and the sociodemographic characteristics of participants. Sense of contribution to society and sense of bonding with the workplace were assessed using the following questions: "Have you ever felt your job contributed to society?" and "Have you ever felt a sense of bonding with your workplace?" Each of the questions was answered on a four-point scale: Always, Often, Rarely, Never.

Severity of psychological distress was assessed using a translated Japanese version of the Kessler Psychological Distress Scale (K10) [17-19]. In the Japanese K10, the Cronbach's alpha coefficient for each measure is 0.911 [20] and its equivalence to the original English version was confirmed [21]. We defined the psychological distress group as those scoring 22 points or higher [22].

To assess job stress factors we used the Japanese Brief Job Stress Questionnaire, which consists of 57 items in 6 domains: quantitative and qualitative workload, job-control latitude, and support from supervisors, co-workers and family [23]. The Japanese Brief Job Stress questionnaire was developed with reference to the Job Content Questionnaire (JCQ) [24] and Job Stress Model by The National Institute for Occupational Safety and Health (NIOSH) [11]. It is the most popular questionnaire for workplace psychological stress-related factors in Japan. The questionnaire includes the following items (for example): (1) have to work hard, (2) must do the work of many, (3) unable to complete the work in time, (4) can work at own pace, (5) can determine own schedule. Four response options are available: Always, Often, Rarely, Never. In the Japanese Brief Job Stress Questionnaire, the Cronbach's alpha coefficient for each stress measure is 0.74 [23]. The items provide continuous variables. We also included age group, sex, job position (managerial or non-managerial), and department as sociodemographic characteristics of participants.

The associations between sense of contribution to society, sense of bonding with the workplace, job stress factors, and sociodemographic characteristics were tested using Spearman's rank-order correlation analyses or Pearson's *χ*^2 ^tests. We performed multiple logistic regression analyses to examine the associations between psychological distress and sense of contribution to society and sense of bonding with the workplace. First, these associations were analysed without adjustment for other variables (model 1). Then, we analysed the associations with adjustment for job stress factors (model 2) or sociodemographic characteristics (model 3). Finally, we analysed the associations with adjustment for job stress factors and sociodemographic characteristics (model 4). Finally, we constructed a model with both sense of contribution to society and sense of bonding with the workplace as explanatory variables. Trends in the odds ratios for sense of contribution to society and sense of bonding with the workplace were tested using constrained linear models [25]. All analyses were computed using SPSS 17 (Chicago, IL) statistical software.

This study was approved by the ethics committee of Akita University Graduate School of Medicine in July 2009. We had the cooperation of the company in this study and obtained written informed consent from the participants.

## Results

The characteristics of the participants are shown in Table 1. Of the 937 respondents with complete data, 75.8% were male and 24.2% were female. The mean age was 37.9 years (SD = 9.6), and 48.3% of respondents were classified as having high psychological distress. More than half the respondents felt some sense of contribution to society (9.7% selecting always, and 49.5% selecting often) and had a sense of bonding with the workplace (8.1% selecting always, and 50.3% selecting often).

**Table 1:** Characteristics of participants (*n *= 937)

Variables	n	%
Sex		

Male	710	75.8

Female	227	24.2

Age		

20-29 years	226	24.1

30-39 years	303	32.3

40-49 years	279	29.8

50-59 years	129	13.8

Job position		

Non managerial	717	76.5

Managerial	220	23.5

Departments		

Sales	95	10.1

System Engineering	727	77.6

Administration	115	12.3

Sense of contribution to society		

Never	75	8.0

Rarely	307	32.8

Often	464	49.5

Always	91	9.7

Sense of bonding with workplace		

Never	92	9.8

Rarely	298	31.8

Often	471	50.3

Always	76	8.1

Mental distress		

High (K10 > 21)	453	48.3

Low (K10 ≤ 21)	484	51.7

Table 2 shows the associations between variables. There was a positive correlation between sense of contribution to society and sense of bonding with the workplace (Spearman's ρ = 0.47). Sense of contribution to society was significantly, positively associated with age group, job position, support from supervisors, support from co-workers, support from family, and job control latitude; and negatively associated with qualitative workload (age group and job position *χ*^2 ^test p < 0.001, other variables Spearman's ρ = 0.07 to 0.23). Sense of bonding with the workplace was significantly, positively associated with age group, job position, support from supervisors, support from co-workers, support from family, and job control latitude (age group, job position and department *χ*^2 ^test p < 0.001, other variables Spearman's ρ = 0.04 to 0.30). Sense of bonding with the workplace significantly differed by each department. The sum totals of "Always" and "Often" were 56.1% for systems engineers, 65.3% for sales staff, and 66.9% for administration staff.

**Table 2:** Correlations between senses of contribution to society and bonding with workplace and other variables

Variables	Sense of contribution to society		Sense of bonding with workplace	
	**Value**	***p***	**Value**	***p***

Sense of bonding with workplace	0.47	< 0.01	-	-

Sociodemographic characteristics				

Sex*	6.36	0.10	6.04	0.11

Age group	0.11	< 0.01	0.09	< 0.01

Job position*	16.0	< 0.01	30.1	< 0.01

Department*	6.68	0.35	3.45	< 0.01

Job stress factor				

Support from supervisors	0.19	< 0.01	0.30	< 0.01

Support from co-workers	0.22	< 0.01	0.28	< 0.01

Support from family	0.14	< 0.01	0.18	< 0.01

Quantitative workload	0.07	0.02	0.04	0.19

Qualitative workload	0.12	< 0.01	0.07	0.05

Job control latitude	0.23	< 0.01	0.23	< 0.01

Spearman's rank-order correlations and Pearson *χ*^2 ^tests (*) were used.

Table 3 shows the results of multiple logistic regression analyses including sense of contribution to society or sense of bonding with the workplace as explanatory variables. A sense of contribution to society was negatively associated with psychological distress even after adjusting for job stress factors or sociodemographic characteristics of participants. After adjusting for both job stress and sociodemographic characteristics, the association became weaker. A sense of bonding with the workplace was negatively associated with psychological distress after adjusting for sociodemographic characteristics of participants. The association disappeared after adjusting for job stress factors.

**Table 3:** Associations between psychological distress and senses of contribution or bonding*

Variables	Model 1		Model 2		Model 3		Model 4	
	**OR (95% CI)**	***P***	**OR (95% CI)**	***p***	**OR (95% CI)**	***p***	**OR (95% CI)**	***p***

Sense of contribution to society								

Never	2.28 (1.53-543)	< 0.01	2.05 (0.99-4.23)	0.05	2.92 (1.53-5.59)	< 0.01	1.99 (0.94-4.19)	0.07

Rarely	1.71 (1.06-2.75)	0.03	1.55 (0.91-2.63)	0.10	1.81 (1.11-2.96)	0.02	1.66 (0.96-2.86)	0.07

Often	1.22 (0.77-1.93)	0.40	1.33 (0.80-2.19)	0.27	1.33 (0.83-2.12)	0.24	1.47 (0.88-2.46)	0.14

Always	Ref	-	Ref	-	Ref	-	Ref	-

	P for trend < 0.001		P for trend = 0.04		P for trend < 0.001		P for trend = 0.06	

Sense of bonding with workplace								

Never	2.47 (1.32-4.61)	0.01	1.36 (0.67-2.78)	0.40	2.49 (1.29-4.79)	0.01	1.24 (0.59-2.62)	0.57

Rarely	2.13 (1.27-3.60)	< 0.01	1.41 (0.79-2.52)	0.24	2.30 (1.33-3.96)	< 0.01	1.39 (0.76-2.55)	0.28

Often	1.49 (0.90-2.46)	0.12	1.26 (0.73-2.18)	0.41	1.52 (0.90-2.56)	0.12	1.21 (0.68-2.13)	0.52

Always	Ref	-	Ref	-	Ref	-	Ref	-

	P for trend < 0.001		P for trend = 0.32		P for trend < 0.001		P for trend = 0.40	

*The associations between psychological distress and senses of contribution to society or bonding with workplace were analysed separately using 4 logistic regression models.

Model 1: Without adjustment.

Model 2: Adjusted for job stress factors (support from supervisors, support from co-workers, support from family, quantitative workload, quality of workload, and job control latitude).

Model 3: Adjusted for sociodemographic characteristics of participants (sex, age group, job position, and department).

Model 4: Adjusted for sociodemographic characteristics of participants and job stress factors.

OR- odds ratio, CI- confidence interval.

Trends on the odds ratios were found for sense of contribution to society (p = 0.01), and sense of bonding with the workplace (p = 0.04) in model 1; in model 2 (p = 0.06, p = 0.87); in model 3 (p = 0.02, p = 0.03) and in model 4 (p = 0.09, p = 0.90). Although the associations between having a sense of social contribution and psychological distress after adjustment for job stress factors (models 2 and 4) weakened, they showed a similar tendency as the results of models 1 and 3, as shown in Table 3.

Table 4 presents the results of multiple logistic regression analysis for models 3 and 4, which include a sense of contribution to society and a sense of bonding with the workplace as explanatory variables at the same time. Among the sociodemographic variables, sex and age group were significantly associated with psychological distress in model 3. In model 4, age group, support from supervisors and family, qualitative workload, and job control latitude were significantly associated with psychological distress.

**Table 4:** Results of models including sense of contribution and bonding*

Variables	Model 3**		Model 4**	
	**OR (95% CI)**	***p***	**OR(95% CI)**	***p***

Sense of contribution to society				

Never	2.27 (1.11-4.65)	0.03	2.03 (0.91-4.49)	0.08

Rarely	1.42 (0.83-2.43)	0.21	1.59 (0.88-2.87)	0.12

Often	1.17 (0.70-1.94)	0.55	1.45 (0.83-2.51)	0.19

Always	Ref		Ref	

P for trend = 0.02			P for trend = 0.09	

Sense of bonding with workplace				

Never	1.71 (0.83-3.54)	0.15	0.94 (0.42-2.11)	0.88

Rarely	1.90 (1.05-3.47)	0.04	1.14 (0.60-2.19)	0.69

Often	1.38 (0.79-2.42)	0.26	1.03 (0.56-1.89)	0.92

Always	Ref		Ref	

	P for trend = 0.03		P for trend = 0.90	

Sex (female vs. male)	0.68 (0.50-0.95)	0.02	0.78 (0.55-1.11)	0.16

Age group				

20-29 years	2.82 (1.74-4.58)	< 0.01	3.19 (1.87-5.44)	< 0.01

30-39 years	1.40 (0.89-2.19)	0.15	1.20 (0.73-1.97)	0.48

40-49 years	1.99 (1.28-3.10)	< 0.01	1.69 (1.04-2.73)	0.03

50-59 years	Ref		Ref	

Job position (non-managerial vs. managerial)	1.01 (0.72-1.41)	0.96	1.07 (0.75 - 1.52)	0.73

Department				

Administration	Ref		Ref	

Sales	0.90 (0.59-1.38)	0.63	1.02 (0.65-1.61)	0.93

System engineering	1.20 (0.68-2.12)	0.53	1.32 (0.71-2.44)	0.38

Support from supervisors			0.88 (0.81-0.95)	< 0.01

Support from co-workers			0.98 (0.89-1.08)	0.67

Support from family			0.84 (0.77-0.92)	< 0.01

Quantitative workload			1.07 (0.99-1.15)	0.10

Qualitative workload			1.19 (1.09-1.31)	< 0.01

Job control latitude			0.84 (0.77-0.91)	< 0.01

*Results are based on multiple logistic regression analysis of models 3 and 4 including both senses of contribution to society and bonding with the workplace as explanatory variables.

**All adjusted variables are shown in the above table.

OR- odds ratio, CI- confidence interval.

## Discussion

Our results indicate that a sense of contribution to society is an explanatory factor relevant to psychological distress that is independent of existing job stress factors. The perceived level of contribution to society was significantly associated with psychological distress, even after adjustment for sociodemographic characteristics or job stress factors. The association was attenuated when both sociodemographic and job stress factors were included simultaneously in a logistic regression model, but the tendency remained. A low sense of bonding with the workplace was also associated with psychological distress after adjustment for sociodemographic characteristics, but no association was found after adjustment for job stress factors. When both sense of contribution to society and sense of bonding with the workplace were entered simultaneously into a logistic regression model, only sense of contribution to society was significantly associated with psychological distress.

The association between sense of bonding with workplace and psychological distress disappeared after adjusting for job stress factors. This indicates that the association was confounded by the effects of job stress factors. It is possible that stressful work environments contribute to both a low sense of bonding with workplace and high psychological distress.

The significant associations between sense of contribution to society and job stress factors suggest that they are related. However, our results indicate that these factors are independently associated with psychological distress.

To our knowledge, in conventional work stress models, a sense of contribution to society has not been considered as a factor affecting worker's mental health. Based on our findings, we suggest that a sense of contribution to society is a factor worthy of consideration in the promotion of mental health in the workplace. As a management strategy, a company demonstrates its business meaning to society as "the company vision" to motivate and encourage employees to do their job. The theory of business management for motivating an individual [26] or the motivation to achieve a goal [27] is put into practice in many companies for the purpose of increasing productivity. Drucker [28] stated that a focus on contribution is the key to effectiveness at work, and a focus on contribution by itself supplies the basic requirements of effective human relations: communication, teamwork, self-development, and development of others. In this paper, we explored the hypothesis that a sense of contribution to society is associated with psychological distress. We further hypothesize that having a sense of contribution to society may boost employee motivation to increase their productivity. Further studies of the association between sense of contribution to society and employee's mental health and productivity would be required to validate this hypothesis.

The sharing of a sense of contribution to society among employees may help to improve their mental health. Shared values in the workplace are similar to the concept of social capital that has been reported in association with mental health of local residents or in workplaces [29,30]. In social capital, there are several categories such as bonding and bridging, and structural and cognitive [31-33]. Shared values are contained in the cognitive dimension, the same as with shared norms. Applying the concept of social capital at work, sharing the meaning of work with the company and employees may help to reduce psychological distress. Meyer argues that commitment in the workplace affects retention, productive action, and health [34]. The relationships between health and sense of contribution to society and bonding with the workplace are expected to be further clarified.

In this study, we used the K10 to measure psychological distress. Untreated psychological distress may lead to premature morbidity and mortality [35]. The associations between psychological distress, the sociodemographic characteristics of participants, and job stress factors in this study were consistent with previous studies. That psychological distress tends to diminish with age is in agreement with previous studies in the Japanese workplace [36,37]. In this study, however, only groups in their thirties were not significantly associated with psychological distress. Mirowsky [38] suggests that the fall of depression in early adulthood and the rise in later life mostly reflects life-cycle gains and losses in marriage, employment, and economic wellbeing. Regarding job stress factors, this study showed that less support from supervisors and one's own family, and more qualitative workload and lower job control latitude were significantly associated with psychological distress. It is known that psychological distress increases with low job control and high workload [7-9]. On the other hand, when social support from supervisors, co-workers and one's own family is higher, psychological distress is lower [10,11]. The occupational stress model of NIOSH identifies the support of one's own family as a factor affecting job stress response [11].

In this study, having a higher sense of contribution to society was significantly associated with age group as well as job position. This suggests that a deep involvement in a person's task through practical business experience or management experience leads to a high sense of contribution to society. We did not investigate the way that employees acquire a sense of contribution to society, or whether it was promoted by the corporation. An alternative explanation is that scores on the sense of contribution to society could be considered a reflection of one's self-justification to continue to engage in current tasks, or could be related to a sense of meaninglessness with their task because of psychological distress. These perspectives need to be examined in future investigations.

The response rate in this study was relatively high (88.1%). Differences in response rates by location and department were not observed (Tokyo 88.8%, Osaka 85.7%, Nagoya 87.0%). Therefore, minimal bias was likely to be introduced through non-response. However, several study limitations need to be considered. Firstly, the sense of contribution to society and sense of bonding with the workplace were assessed with only one question each. Additional research is required to validate the accuracy of these measures, but here we have confirmed their relevance. Secondly, this study was conducted within only one company with a cross-sectional design. The prevalence of psychological distress in this study is higher than that reported by previous research of employees in Japan [39] or in other countries [40-42]. Our results may be influenced by the characteristics of the studied company. Further studies are required to confirm causality between sense of contribution to society and psychological distress in the workplace using a cohort design in a large population. Finally, this was a self-administered survey. Social desirability bias is possible, though because of the nature of the questions asked, considered low. The potential for social desirability bias was minimized by assuring participants that collection and analysis of the questionnaires were performed by external contractors.

## Conclusions

Psychological distress in the workplace was found to be associated with sense of contribution to society. It seems that sense of contribution to society is a factor to be taken into consideration in mental health promotion in the workplace.

## Competing interests

The authors declare that they have no competing interests.

## Authors' contributions

KO, YM, and YK made substantial contributions to the conception and design of the study and were involved in drafting and reviewing the manuscript. KO and YK contributed to the data acquisition process. KO, YM, YK, and KF contributed to the analysis and interpretation of the data. All authors have read and approved the final manuscript.

## Pre-publication history

The pre-publication history for this paper can be accessed here:

http://www.biomedcentral.com/1471-2458/12/253/prepub
